# Hepatitis E Virus Superinfection: an Underrecognized Trigger of Acute Hepatitis B Virus Flare

**DOI:** 10.7759/cureus.13809

**Published:** 2021-03-10

**Authors:** Adham E Obeidat, Gabriel Monti, Wichit Sae-Ow, Hiroko Shinoda, Herbert Lim

**Affiliations:** 1 Internal Medicine, University of Hawaii, Honolulu, USA; 2 School of Medicine, University of Hawaii John A. Burns School of Medicine, Honolulu, USA; 3 Pathology, The Queen's Medical Center, Honolulu, USA; 4 Gastroenterology and Hepatology, The Queen's Medical Center, Honolulu, USA

**Keywords:** hepatitis b infection, hepatitis b flare, hepatitis e virus, liver

## Abstract

Hepatitis E virus (HEV) infection is a significant cause of acute hepatitis in endemic areas, such as parts of Asia, Africa, and Mexico, though HEV prevalence in the United States has been estimated between 6% and 20%. Chronic hepatitis B virus (HBV) infection affects about 1 per 1.4 million people in North America. Although well documented in Asia, HBV flare secondary to HEV superinfection is rarely reported in the United States. Here, we present a case of chronic undiagnosed HBV infection with acute flare secondary to HEV superinfection.

## Introduction

Hepatitis E virus (HEV) infection is a significant cause of acute hepatitis in endemic areas, such as parts of Asia, Africa, and Mexico [1,2]. In these regions, where HEV is endemic, it can account for more than half of sporadic acute cases of hepatitis [2]. Moreover, there are multiple genotypes of HEV, which appear to correlate to infection in different geographic regions. Infections caused by genotype 3 are mostly seen in industrialized counties, such as the United States (US) [3]. HEV prevalence in the US has been estimated between 10% and 20% in the past but this number has decreased to 6% between 2009 and 2010 [4,5]. The wide range of reported seroprevalence is likely due to limited sampling given that testing for HEV is not routinely performed in the US. Also, there is no current US Food and Drug Administration (FDA)-approved commercial test in the US for the detection of HEV immunoglobulin G (IgG), IgM, or RNA [6].

Chronic hepatitis B virus (HBV) infection affects about one per 1.4 million people in North America [7]. Periodic reactivation of HBV, also called chronic HBV flare, causes acute liver injury that may manifest as jaundice, elevated liver enzymes, and elevated HBV serum markers and viral load. It has been well documented that superinfections with HIV and hepatitis D virus (HDV) can lead to chronic HBV flares [7]. However, although well documented in Asia, HBV flare secondary to HEV superinfection is not commonly reported in the US [8]. Here, we present a case of chronic undiagnosed HBV flare secondary to HEV superinfection. 

This case was presented as a poster at the American College of Physicians/Hawaii chapter meeting (virtual platform) February 20, 2021.

## Case presentation

A 34-year-old Micronesian male with a past medical history significant only for traumatic brain injury secondary to a motor vehicle accident was presented to the emergency room with a five-day history of abdominal pain, nausea, vomiting, diarrhea, and one day history of jaundice. The pain was localized to the upper abdomen with no radiation. He also mentioned dark-colored urine, but denied any other urogenital symptoms. He denied any history of fever, decreased appetite, weight loss, or weakness. He denied sick contact, recent travel, or previous episodes of such pain. Patient was seen at a clinic two days before for the same symptoms and was prescribed trimethoprim-sulfamethoxazole, ibuprofen, acetaminophen, and omeprazole with no improvement. Patient denied use of any herbal products or over-the-counter medications. He used to drink alcohol and smoke cigarettes but stopped many years ago. He had no history of illicit drug use, and no family history of gastrointestinal or liver problems. Physical examination showed dry mucous membranes, icteric sclera, yellowish skin, and right upper quadrant and epigastric abdominal tenderness.

Liver function test showed elevated liver enzymes with aspartate aminotransferase 681 IU/L, alanine transaminase 1383 IU/L, alkaline phosphatase 145 IU/L, elevated total bilirubin of 14.2 mg/dL, and direct bilirubin of 9.3 mg/dL. Albumin was 3.6 g/dL and international normalized ratio was 1.5. Complete blood count, kidney function test, and lipase were normal. A CT scan and an ultrasound of the abdomen revealed a mild hepatomegaly of 19.2 cm with a normal echotexture. Cholelithiasis was noted on ultrasound but no inflammatory changes or biliary duct dilation. An MRCP could not be obtained due to the presence of a ventricular-peritoneal shunt. Further evaluation revealed hepatitis B serologies consistent with an acute reactivation of a chronic HBV infection. HBsAg, HBcAb, and HBeAg were positive, while HBsAb, HBcAb IgM, and HBeAb were negative. Quantitative HBV DNA level was more than 91 million IU/mL. Hepatitis C virus Ab, hepatitis A virus Ab IgM, HDV Ab, HSV I&II Ab IgM, Epstein-Barr virus, cytomegalovirus, and toxoplasmosis serologies were all negative. Serum ceruloplasmin, anti-mitochondrial Ab, antinuclear antibody, and rapid plasma reagin were also negative. 

**Figure 1:**
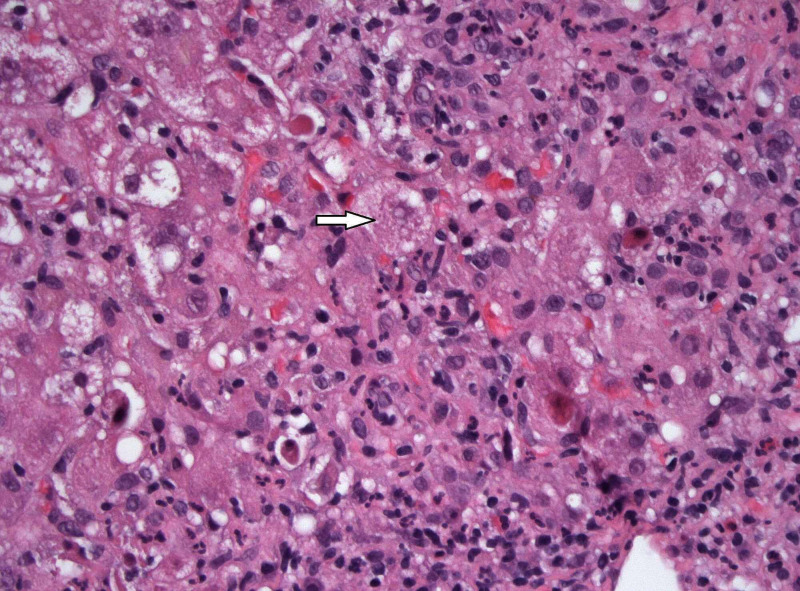
Hematoxylin and eosin stain 400X. The lobules exhibit marked necroinflammatory activity. The hepatocytes show marked reactive changes including prominent swelling and conspicuous acidophil bodies.

**Figure 2:**
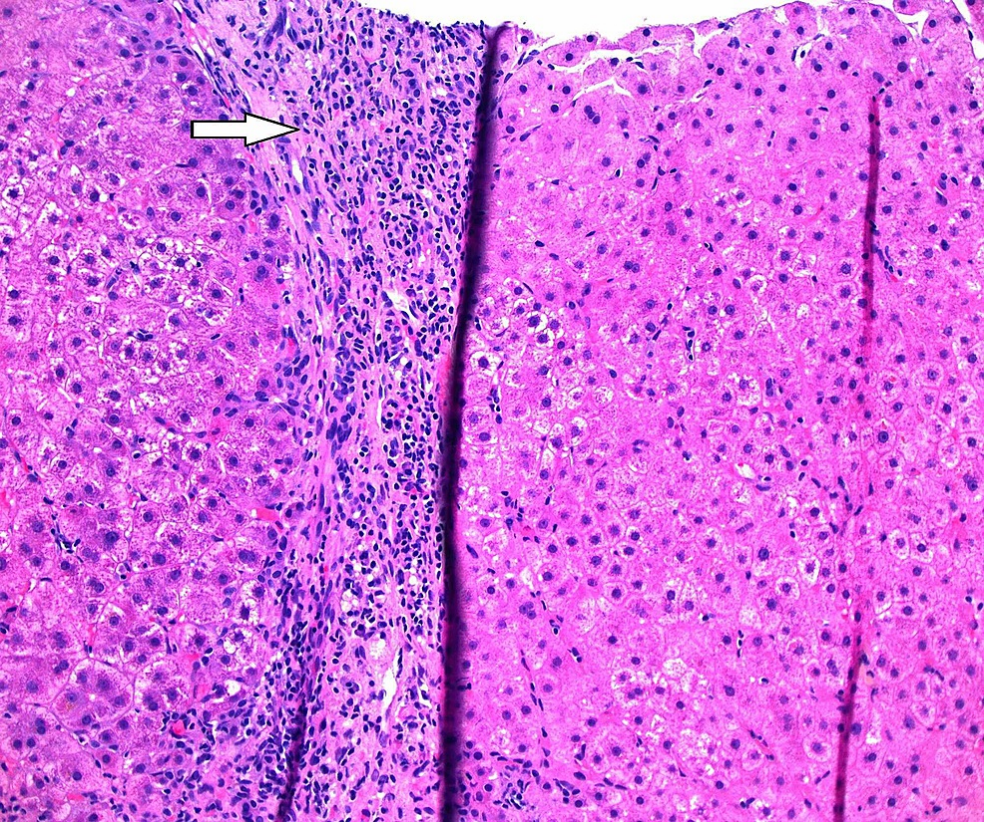
Hematoxylin and eosin 200X shows chronic inflammation in the portal area without significant acute inflammation.

Liver biopsy was done and it revealed inflammatory changes consistent with an acute HBV infection (Figure 1). Entecavir therapy was initiated, and in the course of six weeks, there was a progressive improvement in the patient's liver enzymes, and HBV DNA decreased to 53 IU/mL. However, his total bilirubin remained at 22.1 mg/dL. Due to the lack of improvement of his bilirubin despite improvement of other parameters, other etiologies of hepatitis flare were sought. HEV Ab IgM was done and was positive. Therefore, ribavirin 400 mg bid was initiated and maintained for two months. After four weeks the total bilirubin improved to 11.6 mg/dL, and by the end of the eight-week therapy course, the total bilirubin was 2.8 mg/dL. Liver biopsy was repeated after the completion of therapy and showed chronic inflammation with resolved acute inflammatory changes (Figure 2).

## Discussion

More than 240 million individuals worldwide are infected with chronic HBV. Around 15-40% of untreated patients with chronic HBV infection may develop liver cirrhosis, with an increased risk of liver failure and hepatocellular carcinoma (HCC) [7]. From 565,000 to 1 ,130,000 individuals in the US have chronic HBV infection, and these numbers are higher in communities with large immigrant populations from countries with high prevalence [9]. Chronic HBV infection contributes to at least 50% of HCC cases, and primary liver cancer is the second most common cause of death from cancer in the world [7,10].

The natural course of chronic HBV infection involves fluctuations in transaminase levels and HBV viral load, but it is well established that additional insults to the immune system and to the liver can result in disease flares. For this reason, testing for other etiologies is obtained routinely [7]. HEV infection is not common in western countries including the US, and, therefore, is often not considered among potential insults. Furthermore, in the US, there are no FDA-approved commercial tests for detection of HEV IgG, IgM, or RNA. Thus, the diagnosis of HEV infection is often overlooked or delayed [4,6]. However, a study that was done on 18,695 individuals in the US between 1988 and 1994 showed that as many as 21% of individuals in the general US population had a detectable anti-HEV IgG. This indicates a prior exposure with no antecedent history of hepatitis, which suggests prior subclinical infection, but the number dropped down later on to 6% probably due to limited sampling [4,11,12]. Moreover, the US Drug Induced Liver Injury Network reported that 3% of patients with suspected drug-induced liver injury were likely due to acute HEV since all were anti-HEV IgM positive and some had detectable HEV RNA in their blood [11,13]. HEV causing HBV flares in the US is not commonly documented but has been reported in a 39-year-old Vietnamese woman with chronic stable HBV infection presented with significant flare [14]. 

HEV can be transmitted through different ways. In developing countries, where HEV infection is endemic, the transmission occurs primarily through water contamination, which can lead to either outbreaks or sporadic cases. In developed countries, however, sporadic HEV infection cases have been reported due to the ingestion of undercooked meat [3,14]. Pigs act as a natural reservoir for HEV and can play an important role in the transmission to humans by consuming contaminated meat or via direct contact with animals [15]. We are not sure how our patient acquired the HEV infection but it was likely due to consumption of contaminated food. 

Acute HEV superinfection has been associated with poor outcomes in patients with chronic HBV both worldwide and in the US [8-10]. In a retrospective study that included 228 patients with acute HEV infection with underlying chronic HBV infection done in China, Chen et al. reported that acute HEV superinfection led to poorer outcomes in both cirrhotic and non-cirrhotic patients, especially in patients with underlying diabetes, renal disease, and alcohol consumption [16]. Among patients with acute liver failure in the US, those who were positive for HEV IgG had lower overall three-week survival [11]. Fortunately, our patient did well after treatment with ribavirin, which resulted in significant improvement of his liver enzymes and bilirubin levels. Currently, his chronic HBV infection is controlled on entecavir. 

## Conclusions

Overall, HEV is an often underdiagnosed cause of acute hepatitis in the US. There is a seroprevalence of 6%, and acute infections can be associated with adverse outcomes in patients with underlying liver disease. This case illustrated an immunocompetent patient with chronic HBV infection who suffered an HEV superinfection leading to a delayed response to a flare of chronic HBV infection. Although testing is difficult in the US given the lack of readily available commercial products, HEV infection should be considered in all patients with chronic HBV flare without an identifiable source, even in industrialized nations. We recommend more future studies focusing on the prevalence of HEV in the US due to its effects on pregnant ladies and immunocompromised patients in particular, as well as the general population. 
